# In-Situ Characterization of Pore Formation Dynamics in Pulsed Wave Laser Powder Bed Fusion

**DOI:** 10.3390/ma14112936

**Published:** 2021-05-29

**Authors:** Seyed Mohammad H. Hojjatzadeh, Qilin Guo, Niranjan D. Parab, Minglei Qu, Luis I. Escano, Kamel Fezzaa, Wes Everhart, Tao Sun, Lianyi Chen

**Affiliations:** 1 Department of Mechanical Engineering, University of Wisconsin-Madison, Madison, WI 53706, USA; hojjatzadeh@wisc.edu (S.M.H.H.); qguo46@wisc.edu (Q.G.); mqu22@wisc.edu (M.Q.); escanovolque@wisc.edu (L.I.E.); 2 Department of Materials Science and Engineering, University of Wisconsin-Madison, Madison, WI 53706, USA; 3 X-ray Science Division, Advanced Photon Source, Argonne National Laboratory, Lemont, IL 60439, USA; niranjanparab@gmail.com (N.D.P.); fezzaa@aps.anl.gov (K.F.); 4 Department of Energy’s Kansas City National Security Campus Managed by Honeywell FM&T, Kansas City, MO 64147, USA; weverhart@kcnsc.doe.gov; 5 Department of Materials Science and Engineering, University of Virginia, Charlottesville, VA 22904, USA

**Keywords:** laser powder bed fusion, additive manufacturing, pore, pulsed emission, X-ray imaging

## Abstract

Laser powder bed fusion (LPBF) is an additive manufacturing technology with the capability of printing complex metal parts directly from digital models. Between two available emission modes employed in LPBF printing systems, pulsed wave (PW) emission provides more control over the heat input compared to continuous wave (CW) emission, which is highly beneficial for printing parts with intricate features. However, parts printed with pulsed wave LPBF (PW-LPBF) commonly contain pores, which degrade their mechanical properties. In this study, we reveal pore formation mechanisms during PW-LPBF in real time by using an in-situ high-speed synchrotron x-ray imaging technique. We found that vapor depression collapse proceeds when the laser irradiation stops within one pulse, resulting in occasional pore formation during PW-LPBF. We also revealed that the melt ejection and rapid melt pool solidification during pulsed-wave laser melting resulted in cavity formation and subsequent formation of a pore pattern in the melted track. The pore formation dynamics revealed here may provide guidance on developing pore elimination approaches.

## 1. Introduction

The laser powder bed fusion (LPBF) additive manufacturing (AM) process is a 3D printing technology, which selectively melts powders in successive thin layers to build three dimensional parts directly from digital models without the constraints of traditional manufacturing methods. Currently, the use of LPBF is rapidly growing with multiple industrial applications, such as in the medical, aerospace, defense, and automobile industries [1].

One of the primary distinctions between commercial LPBF systems is the type of laser emission mode employed [2]. In continuous wave LPBF (CW-LPBF) systems, the laser delivers energy continuously without interruption; while in pulsed wave LPBF (PW-LPBF) systems, the laser power is fast modulated to turn on and off repeatedly, delivering energy in pulses [3,4]. The short burst of energy with PW-LPBF creates a melt pool with more flexible control over the heat input, which is highly advantageous for printing finer features such as lattice structures [5]. However, parts printed with PW-LPBF exhibit pores because the pulsated laser can cause instability in the melt pool leading to the formation of pores [6]. Pores are the major defect in parts printed by LPBF AM, which adversely affect the mechanical properties [7], especially fatigue life [8].

While pore formation during the CW-LPBF process has been studied extensively with post-processing diagnostic techniques [9], in-situ X-ray imaging techniques [10,11,12,13,14,15,16] and high fidelity simulations [13], research on pore formation and its underlying mechanisms during PW-LPBF is limited. Therefore, it is important to implement in-situ diagnostic tools, such as state-of-the-art in situ x-ray imaging techniques, to perform fundamental studies on pore formation during the PW-LPBF process in real time.

In this study, we revealed the dynamics and mechanisms of pore formation during the PW-LPBF process by utilizing in-situ high-speed X-ray imaging with 100 ps temporal resolution and ~2 μm spatial resolution. The results of this study are vital for developing processing parameters to mitigate pore formation and therefore improve the mechanical performance and reliability of parts printed using PW-LPBF. In addition, the results of this research may have implications in other areas where pulsated laser is used [17,18,19,20,21].

## 2. Materials and Methods

**Figure 1:**
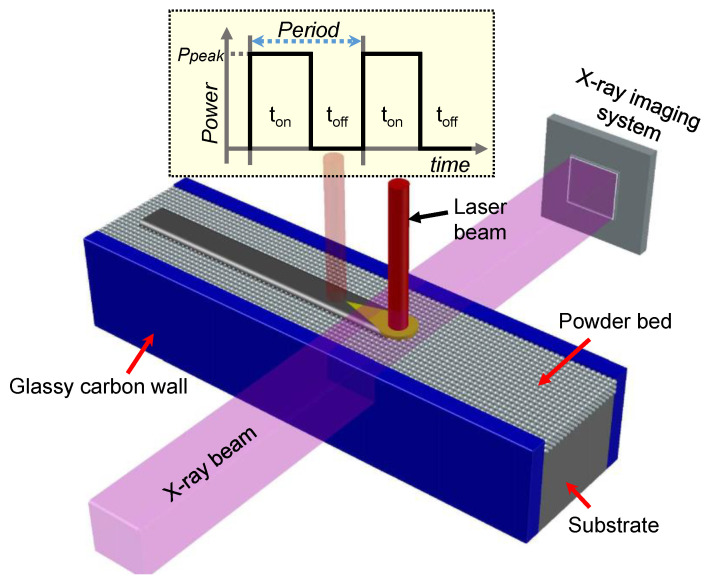
Schematic of the x-ray imaging experiment and the temporal characteristics of the PW-LPBF (pulsed wave laser powder bed fusion) process. The figure shows the temporal characteristic of square shaped pulses used in the experiment and the definition of laser on and off periods. Note that the actual output of the pulse shape may deviate from the perfect square shape specified in the laser control program.

High-speed high-resolution X-ray imaging (at the beamline 32-ID-B of the Advanced Photon Source, Argonne National Laboratory, Lemont, IL, USA) was utilized to probe pore formation dynamics during PW-LPBF in real time [22]. The schematic of the X-ray imaging system is displayed in Figure 1. 

The high-speed X-ray imaging system is composed of a miniature laser powder bed setup which is clamped between two glassy carbon container walls. A pseudo pink X-ray beam, with 1st harmonic energy at (24.7~25.3) keV penetrates through the metal and powder while a downstream detection system converts the transmitted x-ray beam into a visible light image using a scintillator. The converted signal is then recorded by a high-speed camera with a 10× magnification and spatial resolution of approximately 2 μm per pixel [22,23,24,25]. A recording frame rate of 50 kHz was used in this study. The experiments were performed inside a stainless-steel vacuum chamber, under 1 atm argon atmosphere. Ti-6Al-4V and Al6061 plates with the thicknesses of 0.4 and 0.7 mm, respectively, were used as the metal substrates. A layer of Al6061 powder with a thickness of ~100 μm was spread on the top of the Al6061 substrate metal to perform pulsed-LPBF AM experiments. In the experiments with Ti-6Al-4V substrate, no powder was added on the top of the substrate metal.

The key parameters to define the pulse in pulsed laser melting are frequency and laser duty cycle. The frequency (f) is defined as:

(1) $$Frequency=\frac{1}{Period}=\frac{1}{t_{\mathrm{on}}+t_{\mathrm{off}}}$$

where *t*_on_ is defined as the time period when the laser is “on” in each pulse, called the laser-on period, and *t*_off_ denotes the time period when laser is “off” between the end of the pulse and the beginning of the consecutive pulse, called the laser-off period (see the inset of Figure 1). The laser duty cycle is the percentage of the laser-on time in the given modulated period and is defined as:

(2) $$Duty\;cycle=\frac{t_{\mathrm{on}}}{t_{\mathrm{off}}+t_{\mathrm{on}}}\times 100\%$$

An ytterbium fiber laser with the wavelength of 1070 nm, maximum output power of 520 W and a *D*4*σ* diameter of ~100 μm was modulated by a square wave to emit with a given peak power at varying laser frequency (up to 50 kHz) and laser duty cycle (up to 99%) to perform single track laser melting on both powder bed and bare substrate samples. The laser scan velocity was varied from 0.3 to 1.5 m/s in the experiments.

The recorded x-ray images were processed using ImageJ to reduce the noise and enhance the contrast in each frame. The solid–liquid interface was identified in X-ray images by image processing where the image intensity at each pixel of Frame (*i*) was divided by the intensity of corresponding pixel in Frame (*i* + 2), such that the motionless part in the image was converted to blank background [25].

## 3. Results

**Figure 2:**
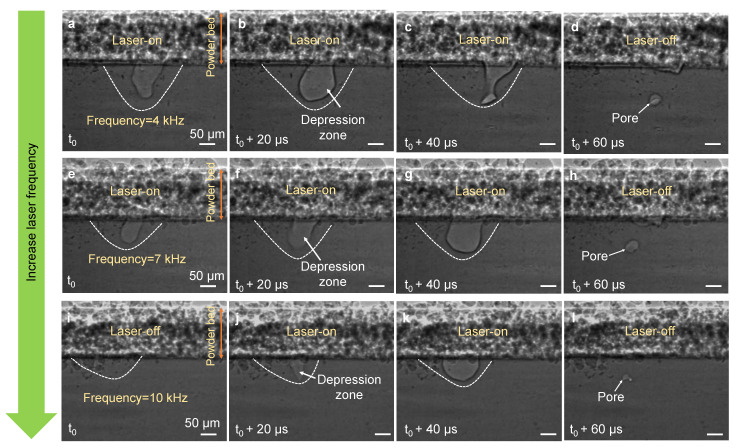
Pore formation under varying laser frequencies at a constant laser duty cycle: (**a**–**l**) Dynamic X-ray images showing pore formation during the PW-LPBF of Al6061 at laser frequencies of 4 kHz (**a**–**d**), 7 kHz (**e**–**h**), and 10 kHz (**i**–**l**) under a laser duty cycle of 50%, a laser power of 470 W and a scan speed of 0.4 m/s. In d, h and l, the melt pool boundary is not indicated to avoid blocking the view of the pores. Note that images do not display the complete duration of one pulse. The compiled movies showing the complete duration of two consecutive pulses are available in the Supplementary Material (Movies S1–S3).

Pore formation during the PW-LPBF process was studied by performing a series of X-ray imaging experiments at a frame rate of 50 kHz under varying laser frequency and laser duty cycle. Figure 2 and Supplementary Movies S1–S3 show pore formation during the PW-LPBF process of Al6061 under varying laser frequency (4, 7, and 10 kHz) and a constant laser duty cycle (50%). Pores are observed to form occasionally via the rapid collapse of the vapor depression at the end of the laser-on period in one pulse which is reminiscent of pore formation at the end of laser track during CW-LPBF AM. The mechanism of pore formation when the laser is turned off at the end of the track has been extensively studied before [11,13,15,22]. Under constant laser duty cycle (while laser power and scan speed are kept constant), the melt pool size is observed to be a function of laser frequency. As the laser frequency increased (from 4 to 10 kHz), a smaller melt pool and therefore a shallower depression zone formed. This caused the formation of pores from vapor depression collapse at the depth closer to the interface between the substrate and the powder layer, as shown in Figure 2.

**Figure 3:**
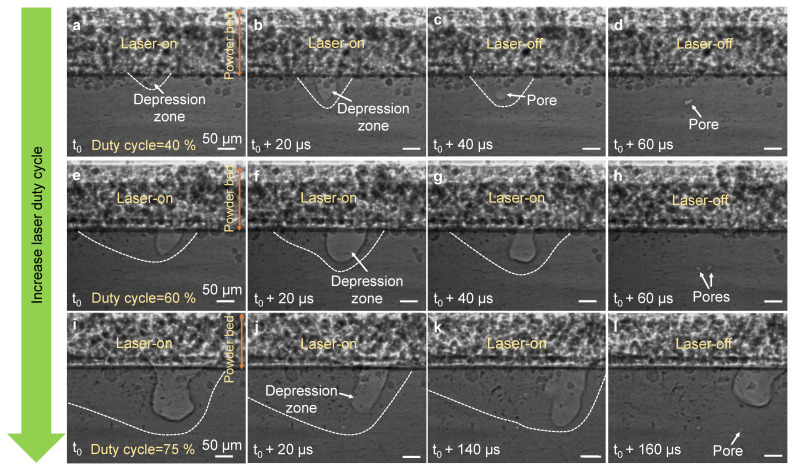
Pore formation under varying laser duty cycles at a constant laser frequency: (**a**–**l**) Dynamic X-ray images showing pore formation during the PW-LPBF process of Al6061 at the laser duty cycle of 40% (**a**–**d**), 60% (**e**–**h**), and 75% (**i**–**l**) at a laser frequency of 7 kHz, a laser power of 470 W and a scan speed of 0.4 m/s. In (**d**,**h**,**l**) the melt pool boundary is not indicated to avoid blocking the view of the pores. Note that images do not display the complete duration of one pulse. The compiled movies showing the complete duration of two consecutive pulses are available in the Supplementary Material (Movies S4–S6).

Similarly, vapor depression collapse at the end of the laser-on period occasionally caused pore formation in the melt pool under a varying laser duty cycle and a constant laser frequency, as shown in Figure 3 and Supplementary Movies S4–S6. The increase in laser duty cycle and therefore longer laser exposure time in these experiments resulted in the formation of a larger melt pool and subsequently the formation of pores at the larger depth relative to the interface between the substrate and the powder layer. From these experimental observations, neither the size nor the number of pores were identified to be correlated with the laser frequency or duty cycle (Figure 2 and Figure 3), which is ascribed to the random pore formation from vapor depression collapse during PW-LPBF.

**Figure 4:**
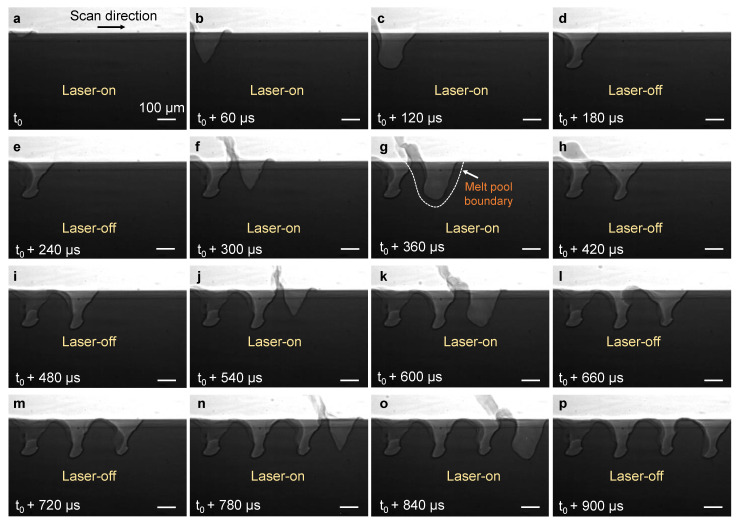
Cavity pattern formation at low laser frequency: (**a**–**p**) Dynamic X-ray images showing formation of cavity during pulsed-wave laser melting of Ti-6Al-4V substrate at a laser frequency of 4 kHz, a duty cycle of 50%, a laser power of 420 W, and a scan speed of 0.8 m/s. Note that some image frames during laser-on time and laser-off time have been skipped. The compiled movie showing the details is available in the Supplementary Material (Movie S7).

Low laser frequency is associated with longer pulse duration $\left(period=\frac{1}{frequency}\right)$ and therefore longer laser irradiation time. To further our understanding of pore formation at low laser frequency, we performed a series of X-ray imaging experiments during pulsed-wave laser melting of Ti-6Al-4V at 4 kHz under a varying laser duty cycle. Figure 4 and Supplementary Movie S7 display the X-ray image sequences during pulsed-wave laser melting of Ti-6Al-4V substrate at a laser frequency of 4 kHz and a duty cycle of 50%. The first frame (*t*_0_, Figure 4a) shows the onset of the laser pulse when laser irradiation starts. First, the laser heating creates a vapor cavity by recoil pressure. After 120 µs, the laser irradiation stops, leading to a rapid freezing of the melt pool and formation of a cavity (Figure 4d). As the consecutive laser pulse begins, the depression zone emerges at a location of ~220 µm distance from the center of the first cavity (*t*_0_ + 300 µs, Figure 4f). The laser irradiation stops again after 120 µs and results in the formation of the second cavity in the substrate (Figure 4h). As the laser moves forward, the cavity formation proceeds and a pattern of cavities is formed in the substrate material (*t*_0_ + 900 µs, Figure 4p).

**Figure 5:**
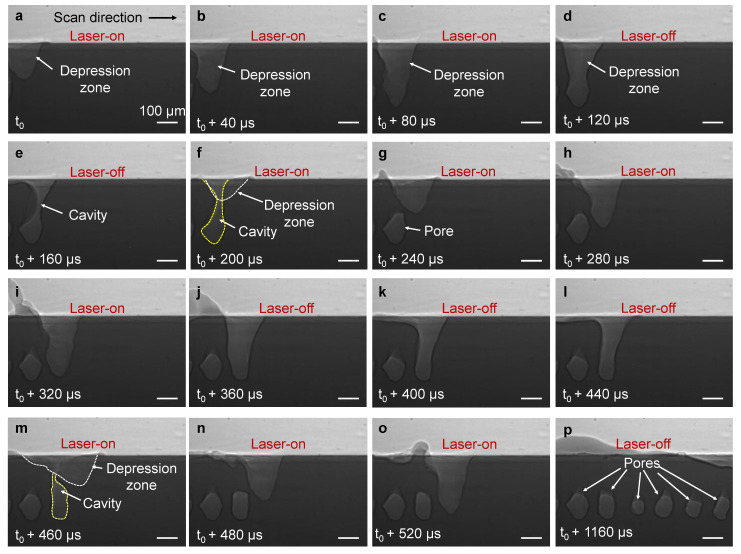
Pore pattern formation from cavities at low laser frequency: (**a**–**p**) Dynamic X-ray images showing the formation of pores from a cavity during pulsed-wave laser melting of Ti-6Al-4V at a laser frequency of 4 kHz, a duty cycle of 60%, a laser power of 470 W and a laser scan speed of 0.5 m/s. The pore formation from cavity in two consecutive pulses is shown in (**a**–**o**). Note that some image frames during the laser-on period and laser-off period have been skipped. The movie showing pore formation dynamics is available in the Supplementary Material (Movie S8).

In the modulated laser, the distance that the laser travels at the time interval between pulses (commonly referred to as point distance) is decreased via the decrease in laser scan speed. This results in the formation of the overlap between melt pools of the consecutive pulses. Figure 5 and Supplementary Movie S8 show an X-ray imaging experiment with pulsed-wave laser melting of Ti-6Al-4V substrate at a laser frequency of 4 kHz, a duty cycle of 60% and a laser scan speed of 0.5 m/s. The cavity forms after the rapid freezing of the melt pool at the end of the laser-on period. As the consecutive pulse begins, the vapor depression emerges at a location where it interacts with the cavity, turning the cavity into a closed pore (Figure 5a–o). As the laser melting continues, a pattern of pores form in the substrate via this pore formation mechanism (Figure 5p).

## 4. Discussion

**Figure 6:**
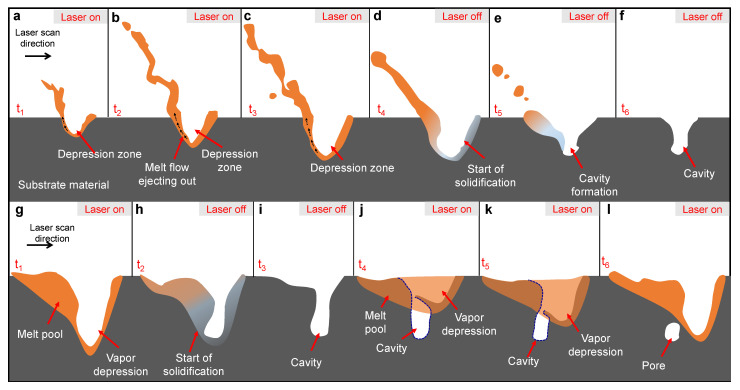
The mechanisms of pore and cavity pattern formation at low laser frequency: (**a**–**f**) Cavity formation mechanism; (**g**–**l**) Mechanism of pore formation from cavity at low laser frequency.

We constructed schematics to illustrate the formation mechanisms of the cavity pattern and pore pattern. The mechanism of cavity pattern formation is displayed in Figure 6a–f. During laser melting, vapor cavities are formed progressively in the substrate material by a strong vaporization-induced recoil pressure. The melt pool that forms in one pulse is observed to be only slightly larger than the vapor depression (as indicated in Figure 4g), appearing to form a layer of liquid around the vapor depression. A strong recoil pressure around the vapor depression pushes the molten metal to move rapidly along the vapor depression walls and ultimately eject away near the rim of the vapor depression in the form of a melt ligament and spatter (Figure 6b,c). As a result, a large amount of liquid metal is ejected away rapidly from the melted area around the vapor depression during laser melting. This phenomenon was recently simulated by a high-fidelity model [13]. As the laser turns off, the temperature around the depression zone decreases abruptly, which results in rapid solidification of the remaining liquid around the vapor depression, especially around the bottom part of the depression zone (Figure 4h and Figure 6d–f).

At the laser parameters setting used here, we did not observe the cavity pattering mechanism in Al6061 substrates. This can be ascribed to the higher thermal conductivity of Al6061 relative to Ti-6Al-4V (90 W/mK compared to 35 W/mK [26,27]). In Ti-6Al-4V, pulsed laser melting results in formation of a small melt pool in each pulse which starts to cool and solidify as soon as the pulse is complete. In Al6061 substrate material with higher thermal conductivity, on the other hand, a larger melt pool with longer solidification time forms in each pulse, which appears like a continuous melt pool by looking at consecutive pulses. As the laser irradiation stops in each pulse, there is sufficient melt around the vapor depression to reverse the direction towards the vapor depression side walls and fill the vapor cavity [28]. Similar cavity formation phenomena may be observed in Al6061 substrate by reducing the pulse duration and increasing the laser power. The mechanism of pore pattern formation from cavity is schematically shown in Figure 6g−l. The first cavity forms as a result of rapid solidification of the melt pool (especially around the bottom part of the depression zone) when the laser is switched off at the end of the laser-on period (Figure 6g–i). With the onset of the consecutive pulse, a new melt pool is created where it connects with the cavity formed in the previous pulse (Figure 6j). As the melted zone moves forward and grows in depth with the laser translating, the upper and middle portions of the cavity are filled with the liquid flowing from the melt pool, and the bottom portion of the cavity remains as a closed pore in the substrate material (Figure 6j–l). This process can continue until a pattern of pores is observed in the substrate material.

## 5. Conclusions

In summary, pore formation dynamics during pulsed wave LPBF AM process were directly observed by utilizing synchrotron x-ray imaging technique. The main conclusions are as follows:

1. The collapse of vapor depression, when laser irradiation stops at the end of the laser-on period in one pulse, was observed to occasionally induce pores during the PW-LPBF process under varying laser frequencies and duty cycles.
2. The melt pool and depression zone size changed with laser frequency and duty cycle. With the increase of the laser frequency or decrease of the duty cycle, the melt pool size and consequently the depression zone size decreased during PW-LPBF.
3. Our experimental observations did not reveal any correlation between the size nor the number of pores and the laser frequency or duty cycle.
4. In the depression/keyhole mode laser melting, at a low laser frequency with large point distance, cavity formation proceeds via the rapid solidification of the thin molten metal layer around the vapor cavity, which subsequently results in the formation of a cavity pattern in the substrate material.
5. In the depression/keyhole mode laser melting, at a low laser frequency with small point distance, the interaction of the cavity with the melt pool in the consecutive pulse results in the formation of closed pores and a pore pattern.
6. The results of this study will help the understanding of the PW-LPBF process and guide the development of processing approaches to mitigate pores.

## Data Availability

Not applicable.

## Supplementary Materials

The following are available online at https://www.mdpi.com/article/10.3390/ma14112936/s1, Movie S1: The dynamics of pore formation during pulsed wave LPBF of Al6061 at a laser frequency of 4 kHz, duty cycle of 50%, laser power of 470 W, and laser scan speed of 0.4 m/s; Movie S2: The dynamics of pore formation during pulsed wave LPBF of Al6061 at a laser frequency of 7 kHz, duty cycle of 50%, laser power of 470 W, and laser scan speed of 0.4 m/s; Movie S3: The dynamics of pore formation during pulsed wave LPBF of Al6061 at a laser frequency of 10 kHz, duty cycle of 50%, laser power of 470 W, and laser scan speed of 0.4 m/s; Movie S4: The dynamics of pore formation during pulsed wave LPBF of Al6061 at a duty cycle of 40%, laser frequency of 7 kHz, laser power of 470 W, and laser scan speed of 0.4 m/s; Movie S5: The dynamics of pore formation during pulsed wave LPBF of Al6061 at a duty cycle of 60%, laser frequency of 7 kHz, laser power of 470 W, and laser scan speed of 0.4 m/s; Movie S6: The dynamics of pore formation during pulsed wave LPBF of Al6061 at a duty cycle of 75%, laser frequency of 7 kHz, laser power of 470 W, and laser scan speed of 0.4 m/s; Movie S7: The dynamics of cavity pattern formation during pulsed-wave laser melting of Ti-6Al-4V substrate at a laser frequency of 4 kHz, duty cycle of 50%, laser power of 420 W, and scan speed of 0.8 m/s; Movie S8: The dynamics of pore pattern formation during pulsed-wave laser melting of Ti-6Al-4V substrate at a laser frequency of 4 kHz, duty cycle of 60%, laser power of 470 W and laser scan speed of 0.5 m/s.
